# Supplementary material for: MAD1 upregulation sensitizes to inflammation-mediated tumor formation
Source: PLoS Genet. 2024 Oct 7;20(10):e1011437. doi: 10.1371/journal.pgen.1011437 (PMC11486420; doi:10.1371/journal.pgen.1011437)
Supplement: S1 Text — (A) Schematic showing targeting strategy for editing MAD1 (encoded by the Mad1l1 gene, left) and edited allele (right). Two gRNAs were used to cut on either side of exon 2, the first coding exon of mouse Mad1l1. The edited allele of Mad1l1 includes a TRE3G promoter and an HA tag after the first ATG, which is in exon 2. (B) Schematic of method used to generate dox inducible HA-MAD1 mice. Cas9, 2 sgRNAs, and donor DNA were injected into C57BL/6 zygotes, which were then transferred to a pseudopregnant mouse. Three founder mice had the intended edit, though 1 founder had an additional 13 base-pair deletion in the intronic region after exon 2. Founder mice were bred with a C56BL/6 mouse and the resulting F1 mice were genotyped to ensure germline transmission of the edit. The schematic was generated in Biorender. (C) Genotyping strategy with three primers used to screen for edited mice. (D) Agarose gel showing successful editing in founder mice. KI = Knock In. The founder with a 13-base pair indel is indicated by KI*. NC = No DNA control. (E) Agarose gel showing germline transmission of the edited alleles in the F1 generation. Fig B. CAG-rtTA3 results in dox-inducible expression of HA-MAD1 RNA. (A) Breeding strategy to produce mice that are doubly heterozygous for HA-MAD1 and CAG-rtTA3 (Jax strain #029627). Doubly heterozygous HA-MAD1KI/+;CAG-rtTA3KI/+ animals were used in B-G. Doubly heterozygous mice were bred with animals heterozygous for CAG-rtTA3 to produce HA-MAD1KI/+;CAG-rtTA3KI/KI animals in E-F. Biorender was used to generate the schematic. (B) qRT-PCR of Mad1l1 mRNA showing substantial increase in Mad1l1 mRNA expression (spliced transcripts) in spleen and fat after 1 week of 625 mg/kg dox feed. (C-D) MAD1 protein expression in mice after 1 week on 625 mg/kg dox feed. Red arrows in immunoblot (C) indicate HA-MAD1. (D) Quantification of MAD1 protein level as assessed by immunoblot. (E-F) Immunoblot (E) and quantitation (F) showing that mice homozygous for CAG-rtTA [file pgen.1011437.s001.pdf]

Supporting Information

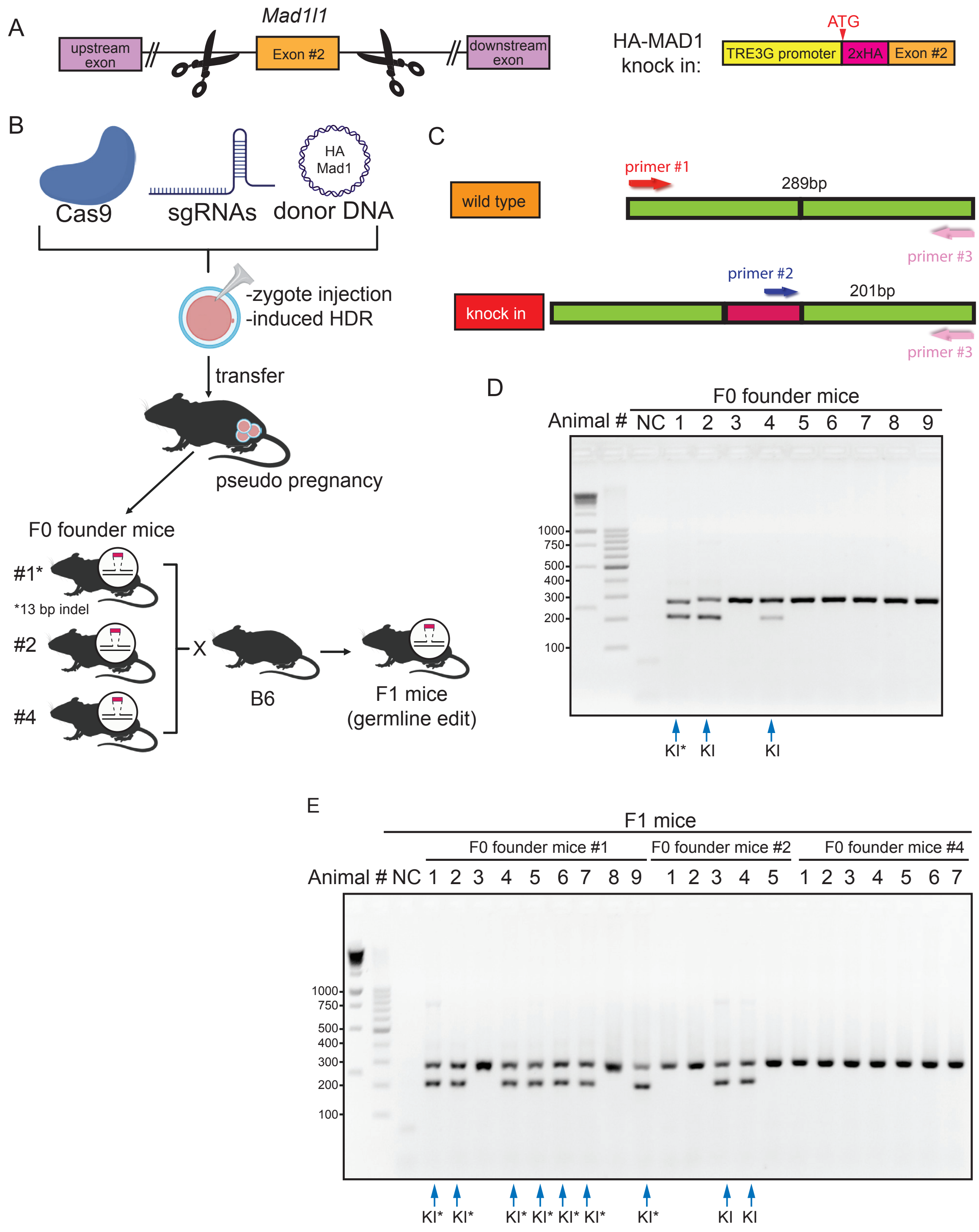

Fig A. Generation and validation of dox-inducible HA-MAD1 mice. (A) Schematic showing targeting strategy for editing MAD1 (encoded by the *Mad1l1* gene, left) and

edited allele (right). Two gRNAs were used to cut on either side of exon 2, the first coding exon of mouse *Mad1l1*. The edited allele of *Mad1l1* includes a TRE3G promoter and an HA tag after the first ATG, which is in exon 2. (B) Schematic of method used to generate dox inducible HA-MAD1 mice. Cas9, 2 sgRNAs, and donor DNA were injected into C57BL/6 zygotes, which were then transferred to a pseudopregnant mouse. Three founder mice had the intended edit, though 1 founder had an additional 13 base-pair deletion in the intronic region after exon 2. Founder mice were bred with a C56BL/6 mouse and the resulting F1 mice were genotyped to ensure germline transmission of the edit. The schematic was generated in Biorender. (C) Genotyping strategy with three primers used to screen for edited mice. (D) Agarose gel showing successful editing in founder mice. KI = Knock In. The founder with a 13-base pair indel is indicated by KI*. NC = No DNA control. (E) Agarose gel showing germline transmission of the edited alleles in the F1 generation.

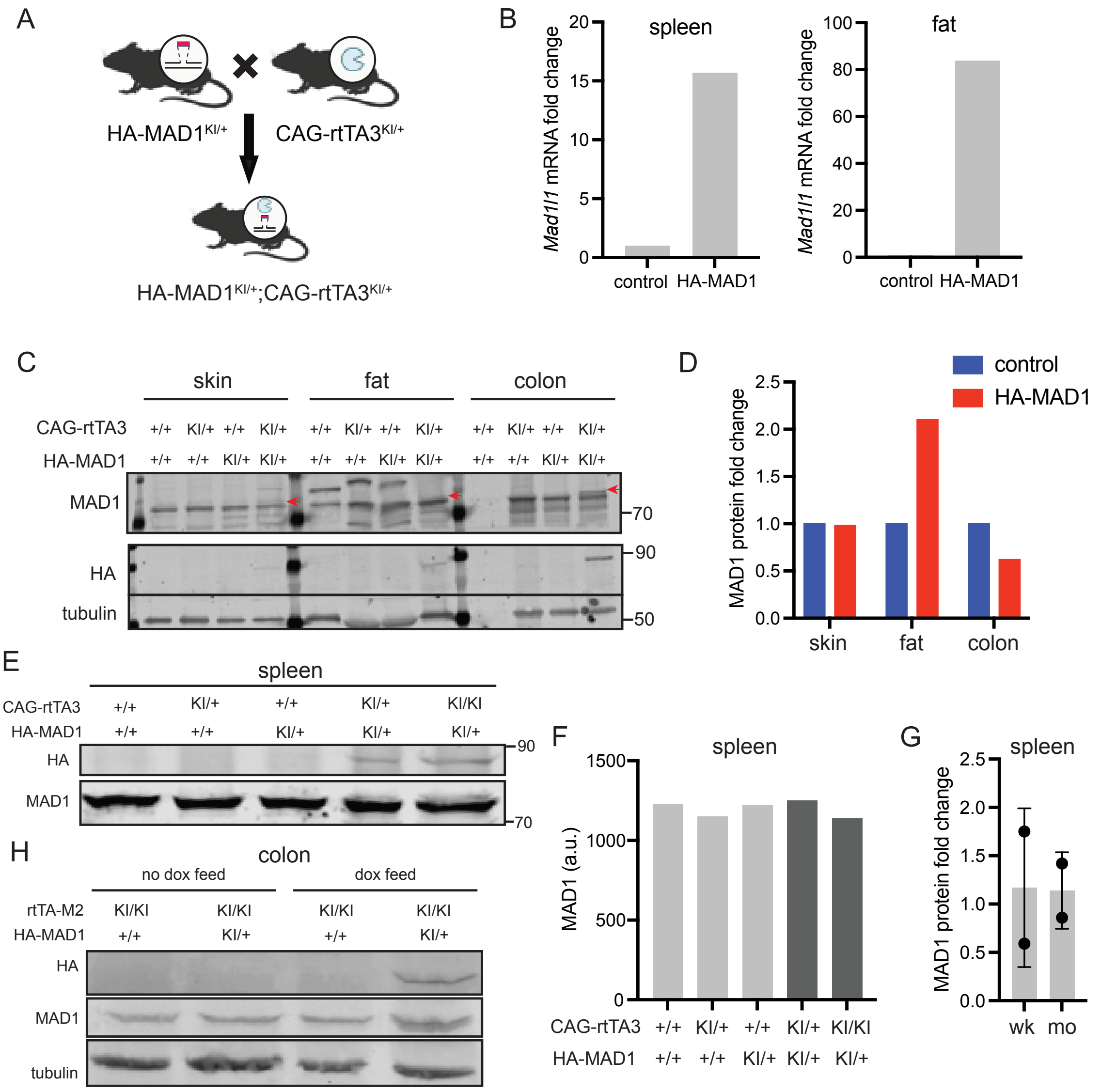

Fig B. CAG-rtTA3 results in dox-inducible expression of HA-MAD1 RNA. (A) Breeding strategy to produce mice that are doubly heterozygous for HA-MAD1 and CAG-rtTA3 (Jax strain #029627). Doubly heterozygous HA-MAD1$^{KI/+}$;CAG-rtTA3$^{KI/+}$ animals were used in B-G. Doubly heterozygous mice were bred with animals heterozygous for CAG-rtTA3 to produce HA-MAD1$^{KI/+}$;CAG-rtTA3$^{KI/KI}$ animals in E-F. Biorender was used to generate the schematic. (B) qRT-PCR of *Mad1l1* mRNA showing substantial increase in *Mad1l1* mRNA expression (spliced transcripts) in spleen and fat after 1 week of 625 mg/kg dox feed. (C-D) MAD1 protein expression in mice after 1

week on 625 mg/kg dox feed. Red arrows in immunoblot (C) indicate HA-MAD1. (D) Quantification of MAD1 protein level as assessed by immunoblot. (E-F) Immunoblot (E) and quantitation (F) showing that mice homozygous for CAG-rtTA3 do not exhibit increased expression of dox-inducible HA-MAD1 relative to mice heterozygous for CAG-rtTA3. (G) Use of dox feed for 1 week and 1 month result in similar levels of MAD1 expression in spleen tissues isolated from HA-MAD1$^{KI/+}$;rtTA-M2$^{KI/+}$ mice. (H) Immunoblot showing expression of HA and MAD1 in control or HA-MAD1 colon with or without dox feed. HA-MAD1 is not expressed in the absence of dox feed.

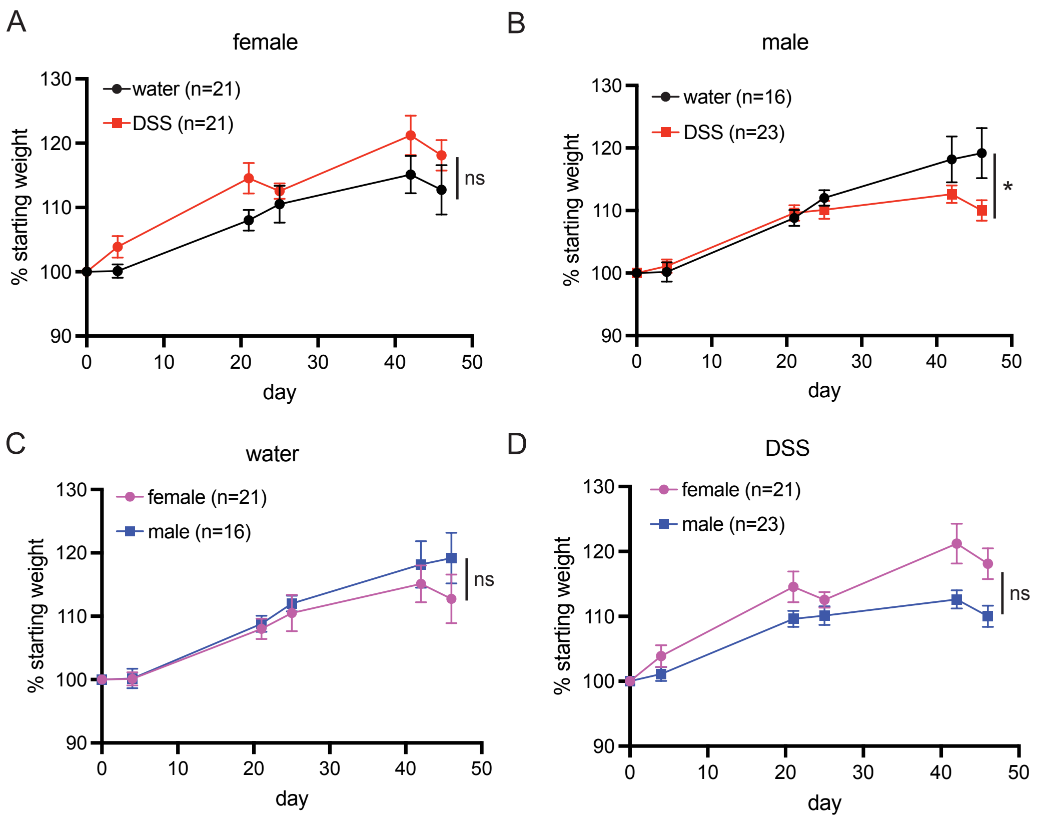

Fig C. Effects of DSS treatment on weight. Mice weight as a percentage of starting weight on day 0 +/- SEM. DSS treatment occurred on days 0-4, 21-25, and 42-46. Mice were weighed at the beginning and end of each cycle. (A) Female mice have similar weight gain in water or DSS treatment groups. (B) DSS treatment impaired weight gain in male mice. (C-D) Data from A-B separated by sex rather than treatment group. ns = not significant; * = $p < 0.05$.

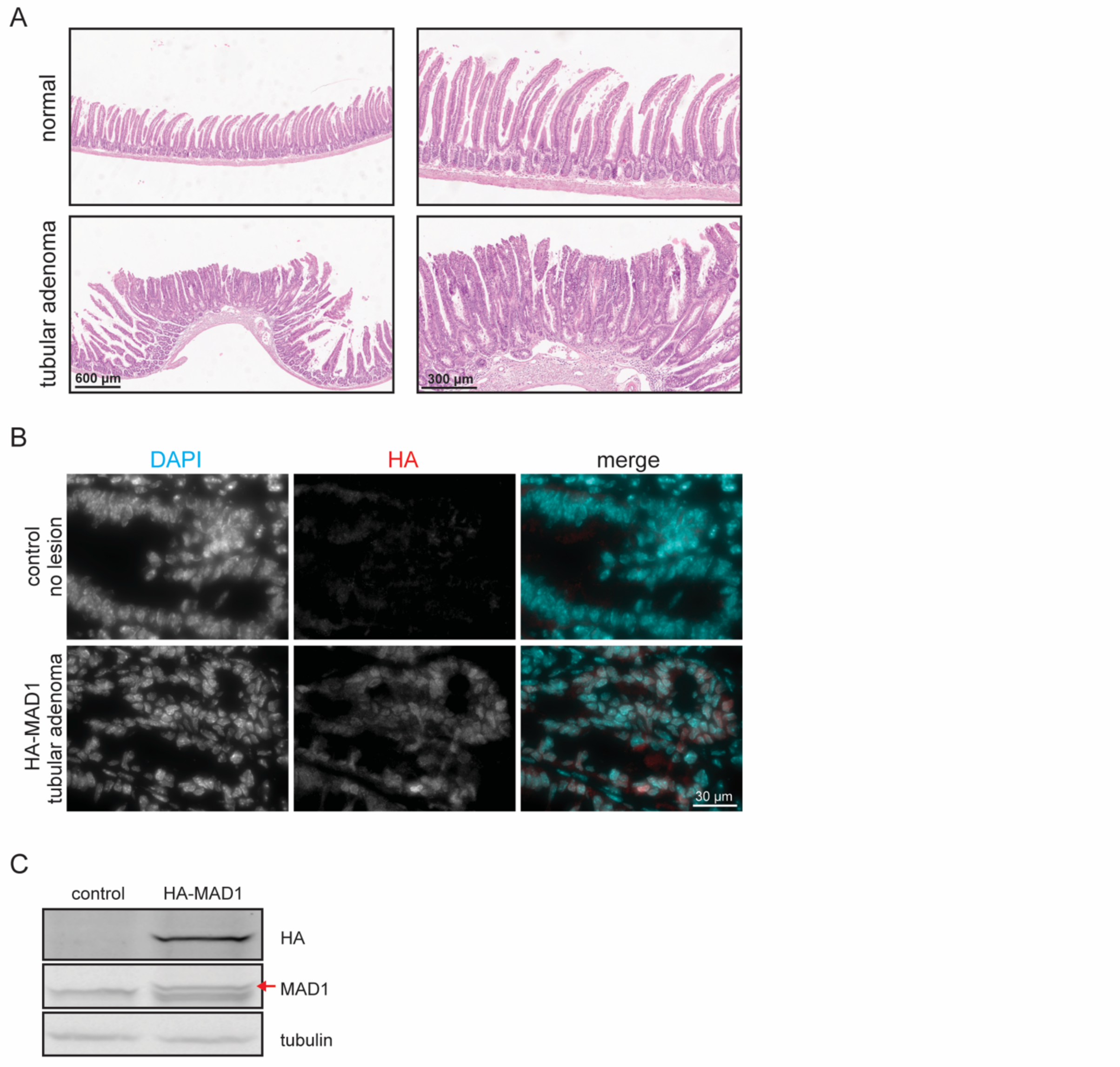

Fig D. HA-MAD1 expression in small intestine. (A) H&E images of normal small intestine and tubular adenoma in HA-MAD1 mouse. Normal indicates normal adjacent tissue. (B) Immunofluorescence showing expression of HA-MAD1 in small intestine tubular adenoma. (C) Dox-inducible expression of HA-MAD1 in small intestine. Tissue for immunoblot was collected after 1 week of dox feed (without DSS treatment). MAD1 upper band (red arrow) corresponds with HA band.

Table A. Homozygous HA-MAD1 mice are embryonic lethal in the absence of dox

genotype	expected (#)	observed (#)	expected (%)	observed (%)
wildtype	17	26	25	38.2
heterozygous	34	42	50	61.8
homozygous	17	0	25	0
total	68	68	100	100

chi square $p < 0.001$

Table B. Potentially inflammation-independent tumors 10 months after cohorts initiated DSS treatment.

genotype	small intestine	prostate	spleen	total
control (n=29)	0	0	0	0
water (n=13)	*0*	*0*	*0*	*0*
DSS (n=16)	*0*	*0*	*0*	*0*
p53 het (n=13)	0	1 (7.7%)	1 (7.7%)	2 (15%)
water (n=6)	*0*	*1 (17%)*	*0*	*1 (17%)*
DSS (n=7)	*0*	*0*	*1 (14%)*	*1 (14%)*
HA-MAD1 (n=28)	1 (3.6%)	1 (3.6%)	0	2 (7.1%)
water (n=12)	*0*	*0*	*0*	*0*
DSS (n=16)	*1 (6.3%)*	*1 (6.3%)*	*0*	*2 (13%)*
