# Supplementary material for: MAD1 upregulation sensitizes to inflammation-mediated tumor formation
Source: PLoS Genet. 2024 Oct 7;20(10):e1011437. doi: 10.1371/journal.pgen.1011437 (PMC11486420; doi:10.1371/journal.pgen.1011437)
Supplement: Attachment — Submitted filename: Response to Reviewers 9-3-24.pdf [file pgen.1011437.s003.pdf]

Reviewer #1: In the current manuscript the authors develop a novel mouse model that has modest overexpression of the important spindle assembly checkpoint protein Mad1. Previous studies have shown that Mad1 is overexpressed in multiple tumor types and there is some evidence that it can induce chromosome mis-segregation as well as destabilize p53, two events that are pro-tumorigenic. In the present study, the authors use CRISPR genome editing to introduce a doxycycline inducible promotor and HA tag into the endogenous Mad1l1 gene locus in mice, such they were able to induce expression by feeding the mice doxycycline. This resulted in ~ 2-fold increase in expression of the Mad1p, which resulted in a decrease in p53 and an increase in chromosome segregation errors. They next treated the animals with a colon-specific inflammatory agent, which induced colon tumors in 31% of the mice, suggesting that overexpression of Mad1 can cause tumors in response to inflammation in immune-competent mice. Overall, the findings are important and will be of interest to the readership of PLOS Genetics, as Mad1 is a critical player in the spindle assembly checkpoint, and there is ample evidence that its mis-expression is correlated with tumorigenesis. This paper is largely the characterization of this mouse, which is necessary before this valuable tool can be utilized by the research community. I am in strong support for publication after the authors address the comments below.

We thank the Reviewer for their positive evaluation of our work, as well as their suggestions for improving it.

General Comments:

1. My main concern with the paper is that the number of tumors formed is small, making it difficult to draw strong conclusions in some of the experiments or to do robust statistics. The authors started out with a reasonable sample size (16 each for control and experimental), but then only a percentage of the mice formed tumors. It would be crazy to repeat the entire study to increase sample size when it is unlikely that any of the major conclusions would change. To address this concern, there are several places where the authors should change their wording to be slightly more cautious on their interpretations-specific examples are listed below.

We have addressed these in the “Specific Comments” section below.

2. A second general comment is that the authors can improve clarity and presentation with better labeling of figures and descriptions in figure legends. Their work is at the intersection of cancer biology and cell biology. Many of the tissue images and the pathology analysis have no arrows or other indicators to draw the reader’s attention to specific areas. In addition, in many places the figure legends are written as results or conclusions rather than stating specifically what is shown in the figure. Again, specific examples will be given

below, but overall, the authors should revisit all of the figures and legends with a focus on improving clarity.

Thank you for calling this to our attention. We have improved the tissue images and figure legends, as detailed below.

Specific Comments:

1. I understand that the authors have genetically engineered the mice with the dox-inducible promotor to overexpress Mad1, but I find the comparison to mice with WT Mad1 to be a bit confusing. The overexpressed Mad1 is also WT- there is just more of it. When someone compares something to WT, my first thought is always a mutant vs. WT gene. Please consider changing the nomenclature for the WT control. Examples I can think of is Mad1 control vs. Mad1-OE or some variation of the control rtTA. These are just suggestions, and I leave it to the authors to choose the clearest option.

Thank you for raising this point. We see how the previous nomenclature could cause confusion and have changed our terminology to “control” vs. “HA-MAD1”.

2. Throughout the paper the authors use the term CIN to describe the defects in chromosome segregation. Technically, they have only showed anaphase defects and have not demonstrated true CIN, which occurs through multiple cell divisions.

Fair point. We have revised the manuscript to be more consistent in our description of the mitotic defects we observed as being consistent with CIN.

3. Please add the specific study number (numbers) for the TCGA data used in Figure 1.

We have added the study identifier to the legend for Figure 1.

4. There is inadequate description of how the increased fluorescence signal was measured. There is no detail in the methods, and the figure legend is insufficient.

Thanks for pointing this out. We have added this under the “Tissue Immunostaining” subheading of the Materials and Methods section.

5. Curious in Fig. 2F why the increased ratio of mRNA/protein is not maintained in the skin- perhaps it is just that there are only two skin samples so insufficient to make a strong conclusion.

Thank you for raising this point. We scored additional skin samples for the revised manuscript. With these additional data points, the increased ratio of mRNA/protein does appear to be maintained in the skin (revised Figure 2F).

6. For Figure 3B, in the merged image in the red channel still looks grey. If it is indeed red, then the images need to be changed to have increased contrast.

Thanks for bringing this to our attention. We have increased the contrast, as suggested.

7. Figure 1D labels are not clear: there are two lanes of the same thing, but the blots are different. Also, for the p53 labels, I am assuming that the two blots are the different antibodies, but then which one is used for quantification in E? And for the IF in F and G- I realize that the layout doesn't fit, but the quantification in F comes from the images in G- thus the data in F needs to be after G. I understand the layout problems- but having figures out of order is confusing for the reader.

We believe the reference here is to Figure 3D. The two lanes represent two animals of the same genotype. We have clarified this in the figure legend. Quantification of p53 was done with the p53 1C12 antibody. This is now indicated in the figure legend. We have rearranged the order of figure panels F and G so that the images precede the quantification.

8. I disagree with the conclusion that the effects of weight are more pronounced in males. The difference comes down to a single measurement at the latest time point; otherwise, the curves are essentially the same. In addition, the difference between males and females on water (C) is the same magnitude as the DSS effect comparison in (D).

We found it striking that while the magnitude of the difference is the same, the direction changed (i.e. male mice show increased weight gain on water, while female mice show a trend toward increased weight gain after DSS treatment). However, we agree that the difference is largely driven by the final timepoint and have softened our conclusion on this point.

9. I appreciate that you started with a good number of mice and ended up with much fewer, but I don't think you have a sufficient number of tumors to conclude that there is significance to the finding that there were more tumors in male mice- especially when you consider that 14% of tumors in p53hets in panel F is not significant- because what it represents is one mouse. Please soften the wording to suggest a trend rather than a strong conclusion.

We intended to indicate that the 1/7 (14%) tumor incidence in p53+/- animals is not significantly different than the 0/16 tumor incidence in control mice. This is now clarified in the legend for Figure 4F. When we compare tumor incidence within our HA-Mad1 cohort, 4/7 male HA-mice developed tumors as compared to 1/9 female mice. Based on a two-tailed chi-squared test, this is a significant difference ($p=0.0488$).

10. In the description of the tumors in Figure 5, it describes the pathology on 5 tumors, but the data from figure 4 say there were 7 tumors. What happened to the other ones?

Figure 5 provides examples of each type of lesion observed. Two mice developed hyperplastic polyps and two mice developed tubular adenomas with high grade dysplasia, but duplicate images were not included in an attempt to avoid the confusion we caused by showing two independent examples of mice with the same genotype in Figure 3D.

11. For the data in Figure 5, you need arrows, arrowheads and other labels to illustrate this figure. This work is important to both cancer biologists, as well as to cell biologists who may have less experience in pathology. Also, the Ki67 staining is very difficult to detect in these images at the presented magnification because it is not all that high contrast. Please consider scaling to enhance the contrast and visibility of the staining.

Thank you for this suggestion. We have remade Figure 5 to include higher magnification views of the regions of interest with arrows and arrowheads to better present our findings.

12. I am unable to discern the differences in localization of the B-catenin staining at this magnification. Please include insets with increased magnification to better illustrate this point.

We have included higher magnification views of the β-catenin staining, as requested. We have also included additional clarification in the legend for Figure 5.

Reviewer #2: This study describes the impact of a modest (2-fold) overexpression of MAD1 in the mouse. Decreased p53 expression and increased chromosomal instability (CIN) are observed in the colon. When combined with exposure to the inflammatory agent dextran sulphate sodium (DSS), 31% of mice develop colon lesions and cancers are observed. More lesions are observed in males. In addition, lesions are observed in tissues (small intestine and prostate) not directly impacted by DSS-induced inflammation.

This is a well controlled study, carefully examining the impact of mild MAD1 overexpression in a novel genetically-engineered mouse model. The manuscript is very clearly written. Once a few issues with the figures and legends have been clarified, I will be happy to recommend publication.

We thank the Reviewer for their positive comments and suggestions.

Figure 3: how low are the p53 levels in the colonic epithelium? No numbers are given in the text. The datapoints in Fig3E-G look close to zero.
MAD1 levels seem to vary significantly from one mouse to another. Eg. Relatively low in the light blue mouse (Fig.3C): do MAD1 expression levels correlate with phenotypes (p53 levels and CIN)? It appears not from panels F and I. Why don't they (in particular for the light blue mouse)?

The values for p53 levels are indeed low (≤12% of control). Specific values are now provided in the source data file. Thank you for bringing our attention to the light blue symbol in Figure 3C. We do not have p53 expression data for that particular animal, preventing correlative analysis in Figure 3G. The revised Figure 3C and 3G contain quantitation of MAD1 and p53 levels from serial sections of the same colons that are appropriately color coded and provide evidence for inverse correlation.

Discussion (p10): 'beyond stabilization of p53'. The authors should expand this section of their discussion, saying how the excess Mad1 protein might perturb chromosome segregation leading to CIN. Similarly, a little more speculation on why tumors are developing in the small intestine/prostate, but only in the mice treated with DSS, will be of general interest.

We have expanded both paragraphs of the discussion related to these topics.

Other points, generally improving figures and legends:

Figure 1: the labelling should be improved and clearly explained in the figure legend. egs. RFS and PPS can be written in full. Q1 (lower quartile) is labelled as 'low' in B and C? Q4 as high? The labelling and description in the legend should be more consistent. HR is ??

We have made the suggested changes to improve clarity. RFS and PPS are written in full. The legend now clarifies that the lower quartile is labeled as low and the upper quartile is labeled as high. HR indicates hazard ratio, which is now indicated in the legend.

Figure 2: can the Mad1 western be improved (Fig.2D)? Particularly in the skin, the Mad1 band is very weak, with several other bands nearby.

We found blotting tissue samples to be substantially more difficult than blotting cell culture extracts, and had particular difficulty blotting the skin tissue samples. Multiple attempts to improve the current version of the blot with the 6 tissue sections run side by side were unsuccessful. We have therefore provided separate blots for each tissue in

Figure 2D in which the Mad1 doublet in the skin is more readily apparent.

Figure 6: Panel D, color should be added to the graph so that individual data-points can be seen (the greys are too dark here, in the mutants). Too many abbreviations are being used here for the general reader. (TA/HGD/HP).

As requested, we have added color to Figure 6D and spelled out tubular adenoma, high grade dysplasia, and hyperplastic polyp.

Figure S2. Western: what are the striking bands observed with anti-Mad1 in the first three fat samples?

We observed these bands in ~50% of the fat samples. They may represent a tissue-specific, alternatively spliced version of MAD1.

Reviewer #3: In this study by Copeland et. al., the authors aimed to investigate the role of Mitotic Arrest Deficient 1 (MAD1L1, MAD1) up regulation, in sensitizing the colon to cancerous lesions. Prior work from this group has shown that MAD1 up regulation in cell lines leads to chromosome instability and destabilization of p53. In this study, the authors successfully generated a mouse model with tet-inducible MAD1 to study the effects of MAD1 up regulation in the colon. They show that a modest 2-fold up regulation in MAD1 leads to chromosome instability and p53 down regulation. MAD1 up regulation, when paired with DSS-induced inflammation, also led to the development of tumorous lesions in the colon, small intestine and prostate. The authors propose that MAD1 up regulation in the colon is sufficient to promote tumorigenesis in inflamed colonic epithelium.
The authors have developed and characterized an elegant tool to generate modest and inducible protein up regulation in-vivo. They have used it to then answer precise questions in a well-designed manner and the conclusions are supported by the data presented. I am overall enthusiastic about the publication of this study in PLOS Genetics. I would only suggest some modifications to the text and the figures for the purpose of making the work accessible to non-experts of the field.

We thank the Reviewer for their positive critique and constructive suggestions for improvement.

Comments

1. In the section where the authors describe the generation of inducible HA-MAD1 mice, the switch between the Rosa26-CAG-rtTA3 and Rosa26-rtTA-M2 systems is very abrupt. A description of these systems is critical to understand their differences in inducing the up regulation of HA-MAD1 mRNA and protein levels.

We transitioned to the Rosa26-rtTA-M2 system because we were unable to reliably achieve inducible expression of HA-MAD1 protein with the Rosa26-CAG-rtTA3 model. We have expanded on our description of these mice in the Results section under the subheading “Generation of dox-inducible HA-MAD1 mice”.

2. All the data shown in figure 2 and the following figures are from mice fed with Dox chow. Though the fact that a homozygous TetO-HA-MAD1 could not be generated suggests that the promoter is suppressed, it would be important to show the basal expression of HA-MAD1 in the absence of Dox chow or, at the very least, explain why there are no experiments where the “no Dox” condition is present.

We agree. We have added Figure S2H demonstrating that HA-MAD1 is not expressed in the absence of Dox feed.

3. Could the authors also comment on why the p53+/- cohort has only one female mouse (figure 4H)? Also, the text states that “14% of p53+/- mice developed tumors”, even though this represents only 1 out of 7 mice and the increase is, according to the author’s analysis, not significant (figure 4F). The authors must tone this statement down.

We were surprised that only 8 of 22 live births in our p53+/- cohort were female. p53 null females often exhibit embryonic lethality due to defects in neural tube closure (Chen et al, *Dev Neurobiol* 2008). However, we did not previously observe reduced births of p53+/- females (Funk et al *Mol Cancer Res* 2000), nor are we aware this has been previously reported. We therefore expect that this was due to the relatively small sample size. 3 female p53+/- animals received DSS treatment in our cohort, but only one survived until the endpoint of 10 months after DSS treatment. Interestingly, the tumor in our p53+/- cohort developed in a male animal, though females heterozygous for p53 are more predisposed than males to develop tumors in human (Chompret et al *Br J Cancer* 2000; Hwang et al *Am J Hum Genet* 2003; Wu et al *Cancer Res* 2006) and mouse (Funk et al *Mol Cancer Res* 2000).

We have toned down the statement by including the number of p53+/- animals that developed tumors (1/7) in the text.

4. In Figure 3D, could the authors please clarify what difference between lanes 2 and 3 are.

The two lanes represent two animals of the same genotype. We have clarified this in the figure legend.

5. In Figure 4H could the authors please comment on whether the difference in incidences of lesions between male and female mice could be because of the extent of DSS induced

inflammation itself? It is important to address this as there is evidence to suggest that DSS affects males more than females (Figure S3 and Chassaing B et. al., 2014).

The increased tumor incidence in male mice could indeed be due to increased inflammation caused by DSS, for the reasons cited by the Reviewer. With respect to Figure S3, we found it striking that male mice show impaired weight gain on DSS, while female mice do not. However, Reviewer 1 pointed out that the difference is largely driven by the final time point, and we have softened our conclusion on this point.

6. The authors claim that the tumors in the small intestine and the prostate in DSS treated HA-MAD1 mice are independent of inflammation. To conclude this, the authors must either show that there are no markers of inflammation present in these tissues (low level inflammation could still be present in the absence of any widespread morphological changes) or tone this statement down.

We meant to indicate that we did not expect DSS to induce inflammation in these tissues based on previous reports. We agree that we have not formally demonstrated that DSS did not induce inflammation in these tissues and have revised our discussion to clarify this point.

7. In the section titled "HA-MAD1 tumors display mitotic defects and decreased p53 expression" the text and data presented indicates that there is not much difference in mitotic defects or p53 levels between tumor and normal colon tissues. The authors should title the section to better represent the data and conclusions presented.

We have changed the section title to "HA-MAD1 tumors and tissue display mitotic defects and decreased p53 expression".

8. Table S2, Could the authors please separate water and DSS treated animals. This would make the data easier to understand/interpret and align better with the text that references this data.

We have updated Table S2 as requested.
